# Detection of Newly Described Astrovirus MLB1 in Stool Samples from Children

**DOI:** 10.3201/1503.081213

**Published:** 2009-03

**Authors:** Stacy R. Finkbeiner, Binh-Minh Le, Lori R. Holtz, Gregory A. Storch, David Wang

**Affiliations:** Washington University School of Medicine, St. Louis, Missouri, USA

**Keywords:** astrovirus MLB1, stools, children, diarrhea, dispatch

## Abstract

The prevalence of the recently identified astrovirus MLB1 in a cohort of children with diarrhea in St. Louis, Missouri, USA, was defined by reverse transcription–PCR. Of 254 stool specimens collected in 2008, 4 were positive for astrovirus MLB1. These results show that astrovirus MLB1 is circulating in North America.

Astroviruses infect a variety of hosts, including humans, turkeys, chicken, cattle, sheep, dogs, cats, deer, ducks, and bats (*1**,**2*). The 8 known human serotypes are genetically closely related. Astroviruses typically cause diarrhea in their hosts; in humans, symptoms usually last 2–4 days (*3*). Children <2 years of age, elderly persons, or otherwise immunocompromised persons are most commonly affected (*3*). Epidemiologic studies suggest human astroviruses 1–8 are responsible for up to ≈10% of cases of acute, nonbacterial diarrhea in children (*4**–**8*).

Recently, a highly divergent astrovirus, referred to as astrovirus MLB1 (AstV-MLB1), was identified in the stool of a 3-year-old boy in Australia (*9*). The entire genome of this novel virus was subsequently sequenced and characterized (*10*). No published reports have described AstV-MLB1 outside of the index case. In this study, we determined the prevalence of this novel virus by reverse transcription–PCR (RT-PCR) screening of stool samples collected at the St. Louis Children’s Hospital in St. Louis, Missouri.

## The Study

Pediatric stool specimens sent for bacterial culture to the clinical microbiology laboratory at the St. Louis Children’s Hospital were analyzed for AstV-MLB1. The Human Research Protection Office of Washington University in St. Louis approved this study. Samples were collected during January through May 2008. Stools were diluted in phosphate-buffered saline at a 1:6 ratio (wt/vol), and total nucleic acid was extracted from 200 µL of each stool suspension by the MagNAPure LC Automated Nucleic Acid Extraction System (Roche, Indianapolis, IN, USA).

Previously described astrovirus primers Mon269 and Mon270 (*11*) frequently have been used for detecting human astrovirus serotypes 1–8 in clinical stool specimens. However, the extensive divergence of AstV-MLB1 to the known human astroviruses rendered these primers unable to amplify AstV-MLB1 (data not shown). Because AstV-MLB1 might represent a new grouping of astroviruses that could include multiple subtypes, we designed primers to conserved regions of the AstV-MLB1 genome to maximize the likelihood of detecting any AstV-MLB1 variant viruses or even other novel astroviruses.

**Figure 1:**
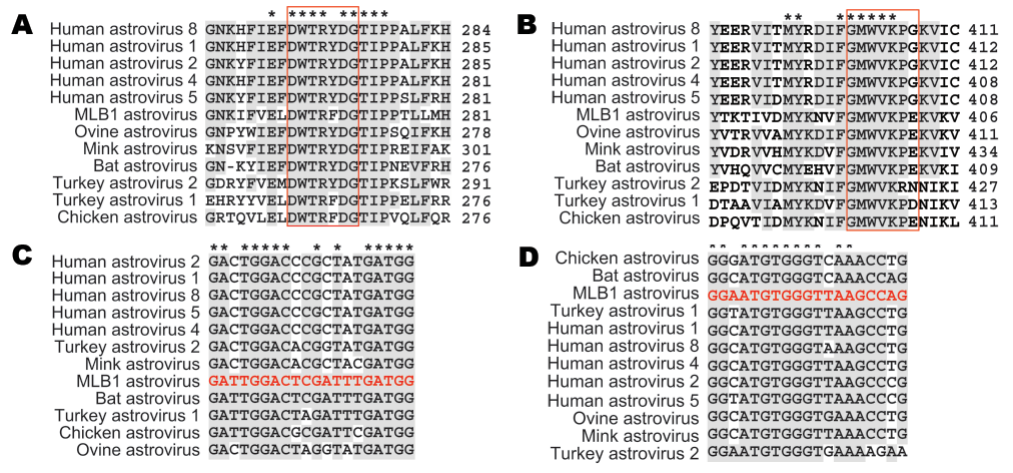
Astrovirus open reading frame (ORF) 1b alignments for design of pan-astrovirus primers. Astrovirus RNA polymerase sequences (ORF1b) were aligned at the amino acid level to define the conserved regions used for the design of primers SF0073 (A) and SF0076 (B). The numbers to the right of the sequences indicate the position of the last amino acid within each ORF1b sequence. Red boxes represent the specific regions that were reverse translated into the corresponding nucleic acid sequences used for the design of SF0073 (C) and SF0076 (D). Red sequences shown in the nucleotide alignments are the actual primer sequences.

We identified conserved regions by using multiple sequence alignments of AstV-MLB1 amino acid sequences to all fully sequenced astrovirus genomes (Figure 1, panels A and B). The corresponding nucleotide sequences for these regions were then aligned to define the most highly conserved regions (Figure 1, panels C and D). Two regions within open reading frame (ORF) 1b were identified that yielded primers SF0073 (5′-GATTGGACTCGATTTGATGG-3′) and SF0076 (5′-CTGGCTTAACCCACATTCC-3′), which are predicted to generate an ≈409-bp product. Control experiments validated this primer pair could detect AstV-MLB1, as well as human astrovirus 1 (Appendix Figure). Given that some of the canonical human astroviruses are identical in the primer binding sites, these data suggest that at least some of the canonical human astroviruses can be detected by the primer pair SF0073/SF0076. In theory, under appropriate experimental conditions, these primers also may be able to detect all other known human and animal astroviruses, although that remains to be experimentally tested. These primers were used with the QIAGEN One-Step RT-PCR Kit (QIAGEN, Valencia, CA, USA) by using the following cycling conditions: 30 min RT step, 94ºC for 10 min, followed by 40 cycles of 94ºC for 30 s, 52ºC for 30 s, and 72ºC for 50 s.

Samples that tested positive with primers SF0073 and SF0076 were then tested in a second round of screening with 2 different primer sets in parallel to determine whether the samples contained canonical human astrovirus serotypes 1–8 or AstV-MLB1. The previously reported Mon269 (5′-CAACTCAGGAAACAGGGTGT-3′) and Mon270 (5′-TCAGATGCATTGTCATTGGT-3′) primers, which generate a 449-bp amplicon, were used to detect canonical human astroviruses (*11*). Another set of primers, SF0053 (5′-CTGTAGCTCGTGTTAGTCTTAACA-3′) and SF0061 (5′-GTTCATTGGCACCATCAGAAC-3′), was designed to exclusively detect AstV-MLB1 and produce a 402-bp PCR product. These primers target a region of the capsid gene. The second round of screening with both sets of primer pairs was performed as described above except that an annealing temperature of 56ºC was used.

**Figure 2:**
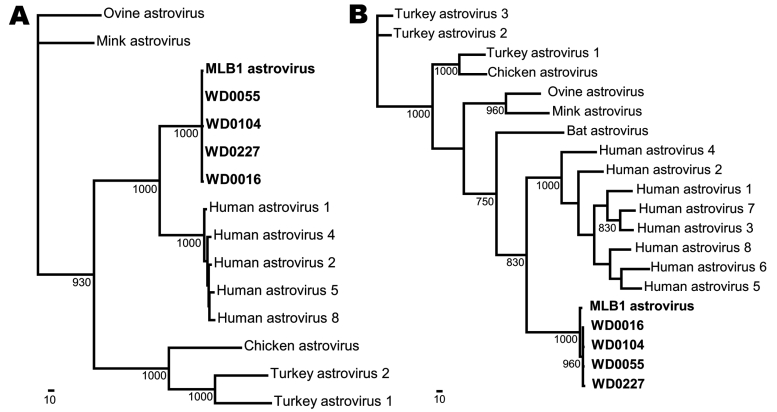
Phylogenetic analysis of astrovirus MLB1 (AstV-MLB1) isolates. A region of the serine protease (A) and the capsid (B) of each virus detected by the AstV-MLB1–specific primers was amplified and sequenced. Multiple sequence alignments were then generated with these sequences and the corresponding regions of known astroviruses using ClustalX (www.clustal.org). PAUP* (Sinauer Associates, Sunderland, MA, USA) was used to generate phylogenetic trees; bootstrap values (>700) from 1,000 replicates are shown. The previously identified AstV-MLB1 isolate (*9*,*10*) and the isolates from this study are shown in **boldface**. Scale bars indicate number of amino acid substitutions per site.

Of 254 stool specimens screened, 9 (3.5%) tested positive in the initial round of screening that used the newly designed pan-astrovirus primers, SF0073 and SF0076. Secondary screening showed that 5 (2% of all samples) were canonical human astroviruses. This probably underestimates the prevalence of the astrovirus serotype 1–8 in the cohort because the initial screening primers were biased toward detection of AstV-MLB1. The remaining 4 (1.6% of all samples) were positive for AstV-MLB1 using primers SF0053 and SF0061. For each of the 4 samples positive for AstV-MLB1, 2 additional fragments were generated by RT-PCR for phylogenetic analysis. A 1,228-bp fragment of ORF1a, which encodes the serine protease, and a 920-bp fragment of ORF2, which encodes the capsid proteins, were amplified using AstV-MLB1–specific primers from each of the 4 samples, designated WD0016, WD0055, WD0104, and WD0227. The primers used for the ORF1a fragment are SF0080 (5′-AAGGATAGTGCTGGTAAAGTAGTTCAGA-3′) and SF0094 (5′-CAAGAGCCTTATCAACAACGTA-3′) and the primers used for the ORF2 fragment are SF0064 (5′-GTAAGCATGGTTCTTGTGGAC-3′) and SF0098 (5′-TGCATACATTTATGCTGGAAGA-3′). The ORF1a fragments (GenBank accession nos. FJ227120–FJ227123) from these samples all shared ≈92% nt identity to the reference astrovirus MLB1 sequence (GenBank accession no.: FJ222451) and 99% aa identity, indicating that most mutations were synonymous. The ORF2 fragments (GenBank accession nos. FJ227124–FJ227127) shared ≈91%–92% nt identity and 95%–96% aa identity to the reference astrovirus MLB1 sequence. The 4 positive St. Louis samples shared ≈99% nt identity to each other. The ORF1a and ORF2 sequences were aligned to other astroviruses for which full genome sequences were available using ClustalX version 1.83 (www.clustal.org); maximum-parsimony trees were generated using PAUP with 1,000 bootstrap replicates (*12*) (Figure 2). The entire genome of one of the isolates, WD0016 (GenBank accession no. FJ402983), was sequenced and had 92.6% identity overall to that of AstV-MLB1 on the basis of a pairwise nucleotide alignment (Table 1).

**Table 1:** Similarity of fully sequenced WD0016 genome to AstV-MLB1*

Genome	Nucleotide identity with AstV-MLB1, %		
	ORF1a (serine protease)	ORF1b (RNA polymerase)	ORF2 (capsid)
WD0016	92.6	93.9	91.9

*AstV-MLB1, astrovirus MLB1; ORF, open reading frame.

Patients with AstV-MLB1–positive stools ranged in age from ≈4 months to 4 years (Table 2). All patients had symptoms of diarrhea at stool collection, except the patient with isolate WD0016, who reported having diarrhea 2 days before stool collection. All specimens were tested for *Escherichia coli*, *Campylobacter* spp., *Yersinia* spp., *Shigella* spp., and *Salmonella* spp. by standard bacterial culture. The WD0227 sample tested positive for *E. coli* O157:H7; the other samples were negative for all bacterial cultures. A pan-viral microarray, the ViroChip (GEO platform GPL 3429; National Center for Biotechnology Information, Bethesda, MD, USA) (*13*), was used to examine whether other viruses were present in the stool of 3 (WD0055, WD0104, and WD0227) of the 4 AstV-MLB1–positive samples for which enough material remained for analysis. WD0055 and WD0104 were negative by array, but WD0227 was positive for rotavirus as determined by the ViroChip.

**Table 2:** Clinical and demographic characteristics of patients with stool samples positive for astrovirus MLB1*

Characteristic	Sample			
	WD0016	WD0055	WD0104	WD0227
Age, m	15	17	4	43
Sex	F	F	M	M
Diarrhea	No*	Yes	Yes	Yes
Other symptoms	Abdominal pain	Vomiting, fever	Fever, seizures, respiratory distress	Fever
Hospitalization	Yes	No	Yes	Yes
Bacterial cultures†	Negative	Negative	Negative	Positive for *E. coli* O157:H7

*Patient had diarrhea 2 days before stool collection but not at collection. †Tests were conducted for *Escherichia coli*, *Campylobacter* spp., *Shigella* spp., *Salmonella* spp., and *Yersinia* spp.

## Conclusions

The newly identified AstV-MLB1 virus was discovered in a stool specimen collected in Melbourne, Victoria, Australia, in 1999. In this study, we describe the detection of AstV-MLB1 in a cohort from St. Louis collected in 2008. This observation provides evidence of AstV-MLB1 outside Australia and suggests that AstV-MLB1 is likely to be globally widespread. In addition, these data demonstrate that AstV-MLB1 is circulating in the human population. The sequence divergence of ≈8% at the nucleotide level between the reference AstV-MLB1 genome and the viruses detected in this study suggests substantial sequence heterogeneity within the AstV-MLB1 group of viruses. Multiple serotypes or subtypes of AstV-MLB1 might exist, as with the canonical human astroviruses. More extensive screening of stool samples with PCR primers targeted toward detection of AstV-MLB1, such as those described here, may provide insight into the true diversity and prevalence of AstV-MLB1–like viruses. Finally, a critical direction for future investigation is determining whether AstV-MLB1, like the canonical astrovirus serotypes 1–8, is a causal agent of human diarrhea, and if so, assessing the extent and severity of disease associated with this virus. Further epidemiologic studies, including both case–control prevalence studies and seroprevalence assays, and efforts to fulfill Koch’s postulates should be pursued.

## Supplementary Material

 Appendix Figure Validation of screening primers SF0073 and SF0076. Primers SF0073 and SF0076 were tested on stool filtrate made from the original AstV-MLB1-positive stool (lane 2) as well as a Human astrovirus 1-positive stool specimen (lane 3) using the QIAGEN One-Step reverse transcription-PCR (RT-PCR) Kit (QIAGEN, Valencia, CA, USA) as described in the text. The products were visualized by electrophoresis on a 1.2% agarose gel. The expected size of the RT-PCR product generated with these primers is approximately 400 bp. Lane 1 shows the Invitrogen (Carlsbad, CA, USA) 100-bp DNA ladder for a size comparison.
