# Supplementary figures and images for: Detection of Newly Described Astrovirus MLB1 in Stool Samples from Children
Source: Emerg Infect Dis. 2009 Mar;15(3):441–4. doi: 10.3201/1503.081213 (PMC2666294; doi:10.3201/1503.081213)

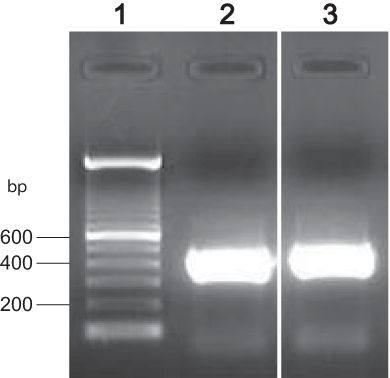

Supplement: Appendix Figure — Validation of screening primers SF0073 and SF0076. Primers SF0073 and SF0076 were tested on stool filtrate made from the original AstV-MLB1-positive stool (lane 2) as well as a Human astrovirus 1-positive stool specimen (lane 3) using the QIAGEN One-Step reverse transcription-PCR (RT-PCR) Kit (QIAGEN, Valencia, CA, USA) as described in the text. The products were visualized by electrophoresis on a 1.2% agarose gel. The expected size of the RT-PCR product generated with these primers is approximately 400 bp. Lane 1 shows the Invitrogen (Carlsbad, CA, USA) 100-bp DNA ladder for a size comparison. [file 08-1213_app-s1.gif]
